# Multi-Head Attention Refiner for Multi-View 3D Reconstruction

**DOI:** 10.3390/jimaging10110268

**Published:** 2024-10-24

**Authors:** Kyunghee Lee, Ihjoon Cho, Boseung Yang, Unsang Park

**Affiliations:** Department of Computer Science and Engineering, Sogang University, 35 Baekbeom-ro (Sinsu-dong), Mapo-gu, Seoul 04107, Republic of Korea

**Keywords:** multi-view 3D reconstruction, attention mechanism, multi-head attention, refiner, object boundary prediction

## Abstract

Traditional 3D reconstruction models have consistently faced the challenge of balancing high recall of object edges with maintaining a high precision. In this paper, we introduce a post-processing method, the Multi-Head Attention Refiner (MA-R), designed to address this issue by integrating a multi-head attention mechanism into the U-Net style refiner module. Our method demonstrates improved capability in capturing intricate image details, leading to significant enhancements in boundary predictions and recall rates. In our experiments, the proposed approach notably improves the reconstruction performance of Pix2Vox++ when multiple images are used as the input. Specifically, with 20-view images, our method achieves an IoU score of 0.730, a 1.1% improvement over the 0.719 of Pix2Vox++, and a 2.1% improvement in F-Score, achieving 0.483 compared to 0.462 of Pix2Vox++. These results underscore the robustness of our approach in enhancing both precision and recall in 3D reconstruction tasks involving multiple views.

## 1. Introduction

In recent decades, the methodologies for creating 3D representations from 2D images have undergone significant changes. The field of 3D reconstruction has seen development of numerous geometric theory-based vision algorithms, such as feature extraction (SIFT) [1], structure estimation using epipolar geometry [2], and model creation via Delaunay triangulation [3]. However, these algorithms consume a considerable amount of computing power, making 3D reconstruction a time-consuming process. The advent of deep learning has shifted these paradigms, offering improvements in both precision and recall, contributing to advancements in 3D reconstruction methodologies. Deep learning has particularly revolutionized the refinement of reconstructed shapes, addressing the challenges posed by the complexity of details and textures inherent in 3D objects.

The attention mechanism is a methodology in artificial intelligence that enables models to focus on the critical aspects of data. This mechanism has been adopted in various forms throughout deep learning networks, with the transformer architecture [4] being one of the most prominent examples that uses the multi-head attention mechanism. The transformer architecture has demonstrated its high performance in both natural language processing (NLP) [4] and computer vision tasks [5]. Given the demonstrated effectiveness of transformers and the precise focus provided by the attention mechanism, this approach is expected to effectively address challenges in 3D reconstruction. Specifically, the original Pix2Vox++ model often struggles with accurate boundary prediction and maintaining high recall rates, particularly for complex geometries. Therefore, we incorporated a multi-head attention mechanism into the refiner module to enhance the model’s capability for focusing on intricate details, thereby reducing boundary prediction errors and improving overall reconstruction accuracy. Multi-head attention enables the model to focus on distinct spatial and contextual features of the input simultaneously, effectively capturing both fine-grained and broader-scale information. This approach is particularly effective for reconstructing complex 3D structures, ensuring accurate representation of intricate boundaries and fine details.

We applied this concept to the existing deep learning 3D reconstruction model Pix2Vox, with the primary contribution of this research being the integration of a multi-head attention mechanism into the refiner module of Pix2Vox++. This enhancement aims to improve the model’s capability to effectively balance the precision and recall of object boundaries, particularly when handling multiple views. Our proposed approach not only enhances boundary accuracy, but also ensures better retention of fine details across different input views, resulting in a superior 3D reconstruction quality. This model takes multiple input images of a single object and reconstructs the 3D object in voxel form. The original architecture of this model employs an encoder–decoder structure along with a U-Net-based refiner. We modified the refiner to incorporate a multi-head attention mechanism for better data flow. With this modification, the modified Pix2Vox++ demonstrates a greater improvement in IoU and F-score values when more than four input images are given. Specifically, our modified model showed fewer errors at the object boundaries and improved recall rates. For example, when using 20-view inputs, our model demonstrated a 2.1% improvement in F-Score, highlighting its robustness in enhancing both precision and recall in multi-view 3D reconstruction tasks.

The remainder of this paper is organized as follows: Section 2 reviews the related work, Section 3 describes the proposed methodology and architecture, and Section 4 presents the experimental results, highlighting how the integration of the multi-head attention mechanism enhances Pix2Vox++ in terms of boundary accuracy and overall reconstruction quality. Finally, Section 5 concludes the paper and discusses future work.

## 2. Related Works

### 2.1. 3D Reconstruction

Comprehensive reviews of 3D object reconstruction approaches can be found in [6]. The 3D reconstruction task can be classified by input types, such as single-view 3D reconstruction and multiview 3D reconstruction, categorized by output format, including voxel grids, point clouds, and 3D meshes. The single-view 3D reconstruction task is a long-established, ill-posed, and ambiguous problem. Before learning-based methods, many attempts have been made to address this issue, such as Shape from X [7], where X may represent silhouettes [8], shading [9], or texture [10]. These approaches require strong assumptions and abundant experience in natural images [11], so they are rarely applied in real-world scenarios. With the advent of learning-based methods, many approaches have achieved strong performances. The 3D Variational Autoencoder Generative Adversarial Network (3D-VAE-GAN) [12] combines a generative adversarial network (GAN) [13] and a variational autoencoder (VAE) [14] to generate 3D objects from single-view images. Marrnet [15] reconstructs 3D objects by estimating the depth, surface normal, and silhouettes from 2D images.

Multiview 3D reconstruction tasks have been studied with algorithm-based methods. Structure-from-motion (SfM) and simultaneous localization and mapping (SLAM) algorithms require a collection of RGB images. These algorithms estimate 3D structures through dense feature extraction and matching [1]. However, algorithm-based methods struggle when multiple viewpoints are widely separated. Furthermore, as the input is discrete information, it cannot offer a full surface of an object, which leads to reconstructing incomplete 3D shapes with occluded or hollowed-out areas. With the learning-based method, Pixel2Mesh [16] is the first to reconstruct the 3D shape in a triangular mesh from a single image. Octree Generating Networks (OGN) [17] uses octree to represent high-resolution 3D volumes with a limited memory budget. Matryoshka Networks [18] continuously decomposes a 3D shape into nested shape layers, which outperforms octree-based reconstruction methods. More recently, AttSets [19] used an attentional aggregation module to automatically predict a weight matrix as attention scores for input features. Both 3D Recurrent Reconstruction Neural Network (3D-R2N2) [20] and Learnt Stereo Machines (LSM) [21] are based on Recurrent Neural Network (RNN), resulting in the networks being permutation variants and inefficient for aggregating features from images of long sequence.

Beyond the domain of 3D reconstruction, there are several additional methods exist for representing 3D objects from 2D images. Novel view synthesis (NVS) is one such method for representing a 3D object. It generates novel photorealistic views by interpolating given 2D images [22,23,24]. Novel view synthesis task has supported 3D reconstruction by generating interpolated images, providing additional data to enhance reconstruction process before Neural Radiance field was introduced. Neural radiance field (NeRF) is a method for synthesizing novel views of complex scenes by optimizing an underlying continuous volumetric scene function using a sparse set of input views [25]. The introduction of Neural Radiance Field marked a turning point, as its exceptional results sparked widespread interest and led a number of following studies. Like the models in 3D reconstruction, some of those variant models [26,27,28] generated a synthesized image from a single image input. Some studies expanded the NVS task into 3D reconstruction. In other words, some studies of NeRFs produced 3D outputs in the form of voxel [29], point cloud, and polygons, while the original NeRF only generated 2D outputs in the form of image and video.

In recent studies, the emphasis has shifted from developing models that perform 3D reconstruction to NeRF models in NVS that are also related to the recently introduced Gaussian Splatting technique [30]. Although we focus on developing a 3D reconstruction model rather than to recent approaches like NeRF, the concept of refining the U-Net structure is generalizable and could be applied across different deep learning architectures, independent of the specific task.

### 2.2. Attention Mechanisms

The general form of the attention mechanism is presented below [31].

(1) Attention=f(g(x)|x)

Here, f(g(x),x) means processing input *x* based on the attention g(x), which is consistent with processing critical regions and obtaining detailed information. Almost all existing attention mechanisms can be written into the above formulation. As attention mechanisms have been researched, a number of variants such as ‘spatial attention [32]: where to pay attention’, ‘temporal attention [33]: when to pay attention’ and ‘channel attention [34]: what to pay attention’ have been proposed. With self-attention and multi-head attention, transformer architecture has been applied in natural language processing tasks, showing significantly improved results [4].

Several studies have used attention mechanism and transformer architecture itself for enhancing 3D reconstruction performance. EVoIT [35] reformulated 3D reconstruction problem as a sequence-to-sequence prediction problem and proposed a 3D Volume Transformer framework inspired from the success of transformer. Differently from previous CNN-based networks, EVoIT has an advantage by unifying the two stage feature extraction and view fusion into a single stage. The aention mechanism allows them to explore the view-to-view relationships from multi-view input images. Self-attention ONet [36] is an enhanced version of ONet [37] incorporating the self-attention mechanism into original 3D object reconstruction model. By employing a self-attention mechanism, models could extract global information, ignore unimportant details, and obtain more consistent meshes. METRO [38] is a mesh transformer framework that reconstructs 3D human pose and mesh from a single input image. By leveraging the transformer, it could simultaneously reconstruct 3D human body joints and mesh vertices. VoRTX [39] model for 3D volumetric reconstruction could retain finer details from fusing multi-view information by performing data-dependent fusion using a transformer.

Inspired by these previous studies, we introduce a multi-head attention refiner that incorporates multi-head attention mechanism into the refiner module to recover finer details from coarse volume.

## 3. Proposed Method

### 3.1. Pix2Vox++ Network Architecture

The Pix2Vox++ [40] network architecture, initially comprising an encoder, decoder, multi-scale context-aware fusion module and a refiner, has been enhanced in this study. The encoder starts by generating feature maps from input images, which are then processed by the decoder to produce coarse 3D volumes. These volumes are further refined by the multi-scale context-aware fusion module, which selects high-quality reconstructions from all the coarse volumes to create a fused 3D volume.

Our study focuses on improving the existing refiner module, which is crucial for correcting inaccuracies in the fused 3D volume but has limitations in capturing intricate object details and maintaining high recall rates. By incorporating a multi-head attention mechanism into the refiner module, we significantly improved the model’s capability to predict precise object boundaries, resulting in more accurate and detailed 3D reconstructions. This integration showcases the effectiveness of attention mechanisms in advancing deep learning models for 3D reconstruction tasks.

### 3.2. Multi-Head Attention Refiner

**Figure 1:**
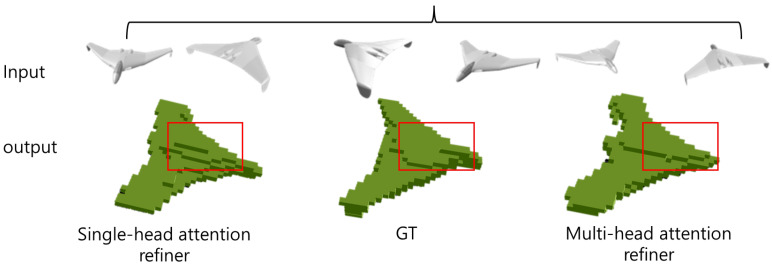
3D reconstruction of an airplane from multi-view and single-view inputs: This figure presents both the angle-specific reconstruction results of an airplane using multi-view inputs and the results obtained using single-view inputs. The comparison demonstrates the effectiveness of our proposed model in translating multi-view 2D input images into accurate 3D objects.

Traditional 3D reconstruction refiners often struggle with maintaining high recall of object boundaries, particularly in complex geometries. The multi-head attention refiner (MA-R) is introduced in this study to address these shortcomings. By integrating the attention mechanism, MA-R selectively focuses on critical regions of the 3D volume, improving precision at object boundaries and enhancing the overall model accuracy. Specifically, we aim to mitigate the boundary prediction errors observed in previous refiners, as shown in Figure 1. The modified refiner, enhanced with multi-head attention, allows for better preservation of detailed information, ensuring that object boundaries are more accurately reconstructed.

MA-R helps tackle one of the most persistent challenges in 3D reconstruction—accurately capturing fine details, particularly at object boundaries, without introducing noise or losing important features. By employing multiple attention heads, MA-R processes the 3D volume at various resolutions, ensuring that both fine-grained and large-scale structures are equally well reconstructed. Focusing on the most informative regions of the volume results in more precise and detailed reconstructions, as demonstrated in our experiments.

Equations (2)–(4) represent the mathematical formulations of the mechanism of self-attention in our multi-head attention refiner. Equation (2) indicates a linear transformation to generate the query vector (*Q*), key vector (*K*), and value vector (*V*). Equation (3) represents the computation of attention weights in self-attention. Finally, Equation (4) denotes the final output, which is the product of the attention weights and the value vectors. This integration of the attention mechanism enables the refiner to focus on the most informative parts of the 3D volume, resulting in a more accurate reconstruction, as shown in our output.

(2) $$\begin{array}{cc} Q,K,V & =\mathrm{Linear}(x) \end{array}$$

(3) $$\begin{array}{cc} g(x) & =\mathrm{Softmax}(QK) \end{array}$$

(4) $$\begin{array}{cc} f(g(x),x) & =g(x)V \end{array}$$

The architecture of our proposed model is fundamentally based on Pix2Vox++, incorporating multi-head attention (MHA) specifically within the refiner. In this architecture, multi-view images are first processed through an encoder–decoder structure to generate coarse 3D volumes. The MHA refiner is then applied to these volumes to refine and enhance the details. The MHA mechanism processes each feature map by computing key, query, and value matrices from the input volume. These matrices are used to calculate the attention weights, determine how much focus each region of the volume should receive based on its relevance to the overall 3D structure.

**Figure 2:**
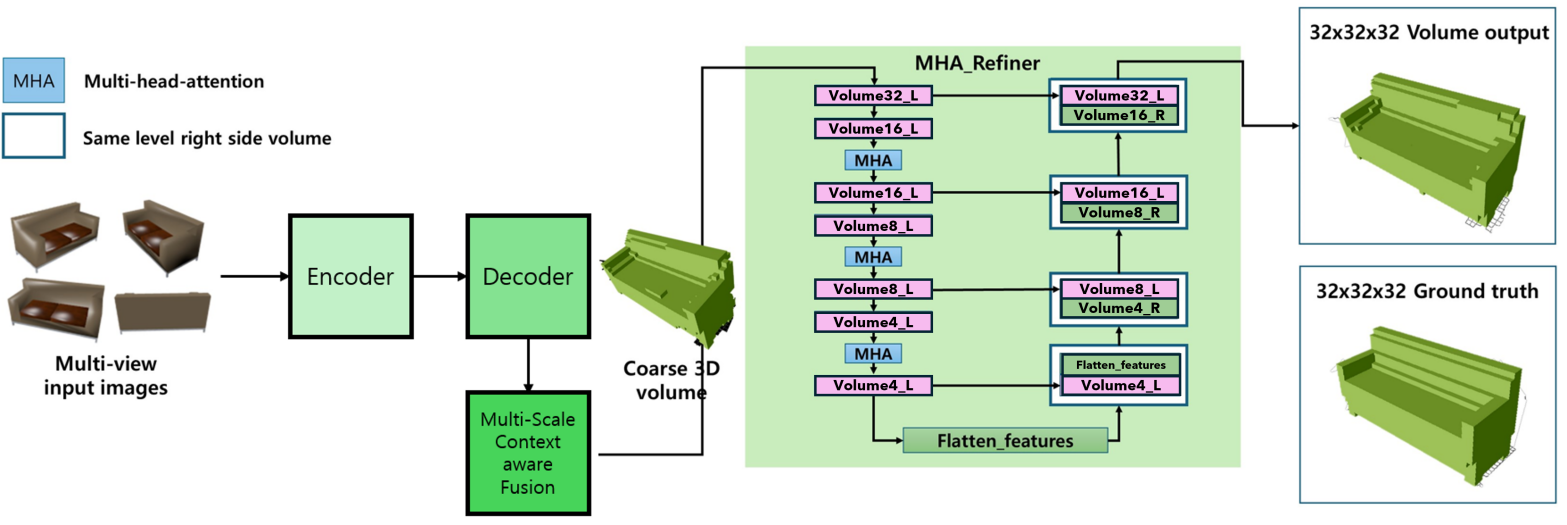
Architectural overview with multi-head self-attention integration in the refiner encoder: This figure demonstrates the integration of multi-head attention within the refiner module of the Pix2Vox++ architecture. The process involves using four-view images and generating a refined 3D volume output of 32×32×32.

Specifically, the MHA refiner employs several heads to provide attention to different aspects of the 3D volume, allowing the model to capture intricate details that may be missed in single-headed attention. The structure of MHA is depicted in Figure 2. Volumes 16-L, 8-L, and 4-L are processed through the MHA, and their outputs are attached to their corresponding volumes of 32-R, 16-R, and 8-R, respectively. This multi-scale refinement process allows the MHA to focus on fine-grained details at multiple resolutions, ensuring that the final volume output of $32^{3}$ retains high levels of detail and accuracy.

**Figure 3:**
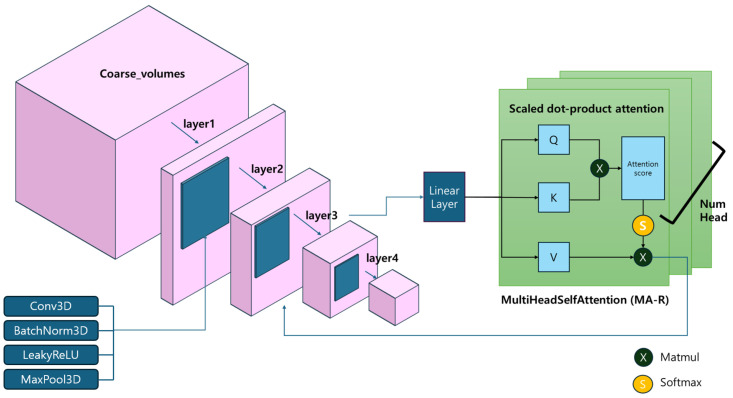
Integration of multi-head self-attention post layer 3: This figure delineates the specific segment within the MA-R refiner where the multi-head self-attention mechanism is employed. It emphasizes the transformation process from the input of the third layer to its output, which then serves as the input for the subsequent fourth layer. This portrays the layered sequential processing and the attention-based enhancement applied within the refiner’s encoder, underlining the effectiveness of the multi-head self-attention in refining 3D reconstruction outputs.

Figure 3 describes how multi-head self-attention is integrated within the encoder section of the refiner. It focuses on the transformation process following layer 3, leading up to the input for layer 4, emphasized by the application of multi-head self-attention. Each layer initially processes the feature map through the convolution 3D layer, batch-normalization 3D layer, LeakyReLu as the activation function, and max pooling 3D layer. In the post-processing layer, the volume undergoes a linear transformation to set the query (Q), key (K), and the value (V). The attention score is generated by the multiplication of the matrix of Q and K. These scores, once normalized with a softmax, stabilize the weights, and their multiplication with V produces an output of the original size, which is fed to the subsequent layers. A unique feature of this model is the repetition of internal attention that extends over several heads.

In the Section 4, the intersection over union (IoU) and F score will be used as quantitative metrics to demonstrate the effectiveness of the proposed method. Other models will also be compared, with a comparison to Pix2Vox serving as an ablation experiment. Additionally, reconstructed 3D objects will be presented as qualitative results.

## 4. Experiment

### 4.1. Datasets

The ShapeNet dataset [41] is a comprehensive and widely used collection of 3D CAD models, organized based on the WordNet taxonomy. It is renowned for its large scale and diversity, encompassing a wide array of object categories, making it a standard benchmark in the field of 3D object reconstruction. ShapeNet provides richly annotated 3D CAD models that are crucial for training and evaluating 3D reconstruction algorithms. In this study, we utilized a subset of the ShapeNet dataset, consisting of approximately 44,000 models across 13 major categories. This selection is aligned with the datasets used in 3D-R2N2 [20], ShapeNetRendering, and ShapeNetVox32. The choice of this subset was driven by our aim to ensure compatibility and facilitate a direct comparison with existing studies, particularly those that have employed 3D-R2N2, a well-established framework in multi-view 3D reconstruction. By using this subset, we aim to benchmark our proposed method against established performances in the field, ensuring that our findings are both relevant and comparable within the current research landscape.

### 4.2. Evaluation Metrics

To evaluate the quality of the proposed methods, we binarized probabilities at a fixed threshold of 0.3 and calculated IoU as a similarity measure between ground truth and prediction.

(5) $$\mathrm{IoU}=\frac{\sum_{i,j,k}I\left({\hat{P}}_{i,j,k}>t\right)I\left({\hat{P}}_{i,j,k}\right)}{\sum_{i,j,k}I\left[I\left({\hat{P}}_{i,j,k}>t\right)+I\left(P_{i,j,k}\right)\right]}$$

where ${\hat{P}}_{i,j,k}$ and $P_{i,j,k}$ represent the predicted occupancy probability and the ground truth in the (i,j,k) voxels, respectively. I(·) is an indicator function and *t* denotes a threshold. Higher IoU values correspond to a better reconstruction accuracy.

We calculate the F-Score as an additional metric to evaluate the performance of 3D reconstruction results. The F-Score represents the harmonic mean between precision and recall, and is defined as follows:

(6) $$F-\mathrm{Score}\left(d\right)=\frac{2\cdot P(d)\cdot R(d)}{P(d)+R(d)}$$

where P(d) and R(d) are the precision and recall for a given distance threshold *d*, respectively. These are computed by:

(7) $$P\left(d\right)=\frac{1}{n_{r}}\sum_{r\in R}\left[\min_{g\in G}∥g-r∥<d\right]$$

(8) $$R\left(d\right)=\frac{1}{n_{g}}\sum_{g\in G}\left[\min_{r\in R}∥g-r∥<d\right]$$

where R and G denote the reconstructed and ground truth point clouds, respectively, and $n_{r}$ and $n_{g}$ represent the number of points in R and G, respectively. We used the Marching Cubes algorithm to extract the object surface from the reconstructed voxel. We sampled 8192 points from the surface to compute the F-Score between the prediction and ground truth [42]. Higher F-Scores indicate better reconstruction quality.

### 4.3. Implementation Details

We trained the proposed methods with batch size of 64 using 224×224 RGB images as the inputs. The output data are $32^{3}$ voxels. We implemented our networks in PyTorch 2.4.0+cu121 [43] and trained Pix2Vox++/A using the Adam optimizer [44] with $\beta_{1}$ of 0.9 and $\beta_{2}$ of 0.999. The initial learning rate was set at 0.001 and decayed by 2 after 150 epochs. We trained the networks for 250 epochs, while multiscale context-aware fusion does not apply in single-view reconstruction tasks. All of the experiments were conducted using an NVIDIA A6000 GPU (NVIDIA Corporation, Santa Clara, CA, USA) on a server provided by Sogang University.

### 4.4. Results

#### 4.4.1. Quantitative Results

This improvement can be attributed to the ability of the MA-R to selectively focus on essential geometric details while minimizing the influence of noise. The multi-head attention mechanism prioritizes high-frequency features, such as sharp edges and object boundaries, which are crucial for preserving the integrity of the 3D reconstruction. Conversely, irrelevant or redundant low-frequency information that could introduce noise is suppressed through the attention mechanism.

As the number of input views increases, the network is better able to refine the object by distinguishing between fine details and noise, leading to more accurate reconstructions, particularly in complex geometries. The improved capability to focus on relevant details directly enhances the model’s performance, resulting in increased accuracy, as evidenced by higher IoU and F-Score metrics.

#### 4.4.2. Qualitative Results

**Figure 4:**
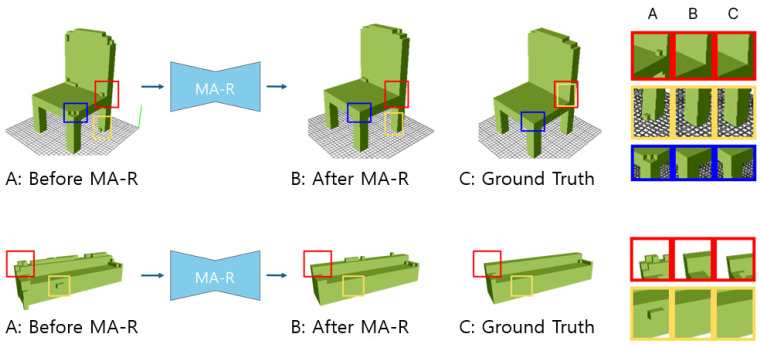
Qualitative demonstration of MA-R performance: Panel A shows the 3D volume before the application of MA-R, Panel B presents the 3D volume after MA-R processing, and Panel C represents the ground truth. The red and yellow boxes highlight areas that were erroneously reproduced in the single head model but have been correctly removed after MA-R, while the blue box indicates a region that was initially missing and has been adequately filled in after MA-R refinement.

**Figure 5:**
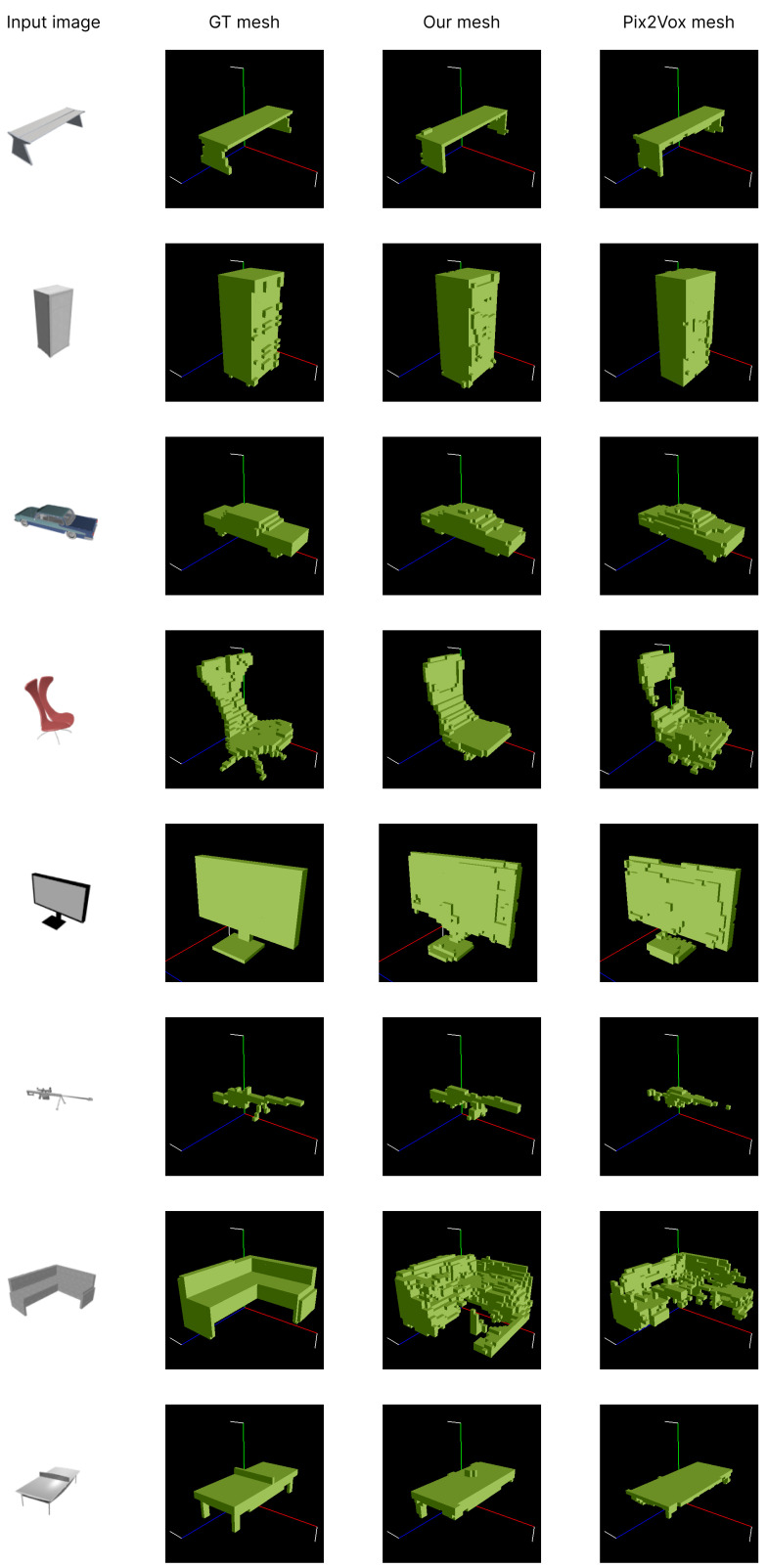
Ground truth meshes and reconstruction results of our model and Pix2Vox++ for various objects: Arranged from top to bottom are the results for bench, cabinet, car, chair, monitor, rifle, sofa, and table.

We also performed a qualitative evaluation of our proposed method. Figure 4 and Figure 5 present the results of this evaluation.

In Figure 4, we demonstrate the qualitative evaluation of MA-R performance using the sofa and chair datasets. The improvements made by MA-R are emphasized by using color-coded boxes. The red and yellow boxes indicate that the voxels that were present but did not exist in the ground truth have been removed after MA-R application. The blue boxes show areas where parts that were missing in the initial model but present in the ground truth have been improved, resulting in higher-fidelity reproduction. This illustrates the superiority of our MA-R in correcting both deficiencies and excesses in the model, thus enhancing overall accuracy.

In Figure 5, we compare our final MA-R output result with the ground truth meshes and the results from Pix2Vox++. In the case of a chair (as shown in the fourth row of the table), unlike the results from Pix2Vox++, we did not meet the problem of having holes in the middle of the object. In additional comparison examples, it is clear that our outputs demonstrate a higher fidelity to the ground truth mesh compared to the outputs obtained from Pix2Vox++.

## 5. Conclusions

The MA-R method has demonstrated significant improvements in boundary prediction and overall reconstruction accuracy for 3D reconstruction. This is supported by the results in Table 1 and Table 2, where our method outperforms the baseline approaches in terms of both IoU and F-Score across multiple view settings. Specifically, at 20 views, our method achieves an IoU score of 0.730 and an F-Score of 0.483, compared to Pix2Vox++’s 0.719 and 0.462, respectively. However, the increased computational complexity of the multi-head attention mechanism, particularly when applied to high-resolution 3D volumes, remains a limitation. This complexity can lead to longer processing times and higher memory consumption, potentially limiting the method’s scalability for real-time applications or with very large datasets. Future research will focus on refining the refiner module, experimenting with larger volumetric data beyond the current 32×32×32 resolution, and exploring more efficient data representations, such as tri-planes, to achieve a better performance with reduced computational costs.

Despite these challenges, the MA-R method holds great promise for advancing the field of 3D reproduction. It effectively addresses persistent issues in multi-view 3D reconstruction, particularly the challenge of maintaining high fidelity at object boundaries. Future research will also focus on improving the performance of the method when applied to larger and more complex 3D volumes, ensuring that the method scales effectively for more detailed reconstructions. By integrating MA-R with emerging techniques such as neural rendering or photogrammetry-based approaches, we can further enhance its capability to handle complex geometries and large-scale environments.

Additionally, refining the balance between noise suppression and detail preservation remains an important area of exploration. While MA-R has shown encouraging results, more sophisticated approaches to identifying and preserving fine details, while minimizing noise, could lead to even more accurate 3D reconstruction. As 3D reconstructions continue to evolve, improving scalability and fidelity for larger, more intricate reconstructions will be key for developing robust, high-performance solutions.

## Figures and Tables

**Table 1:** Quantitative results of multi-view 3D object reconstruction on ShapeNet at $32^{3}$ resolution, with mean IoU for all categories.

Methods	1 View	2 Views	3 Views	4 Views	5 Views	8 Views	12 Views	16 Views	20 Views
3D-R2N2	0.560	0.603	0.617	0.625	0.634	0.635	0.636	0.636	0.636
AttSets	0.642	0.663	0.670	0.675	0.677	0.685	0.688	0.692	0.693
Pix2Vox++	**0.670**	**0.695**	**0.704**	**0.708**	0.711	0.715	0.717	0.718	0.719
Ours	0.636	0.681	0.699	**0.708**	**0.713**	**0.721**	**0.726**	**0.729**	**0.730**

**Table 2:** Quantitative results of multi-view 3D object reconstruction on ShapeNet at $32^{3}$ resolution, with mean F-Score for all categories. **Bold** values indicate the highest performance in each category.

Methods	1 View	2 Views	3 Views	4 Views	5 Views	8 Views	12 Views	16 Views	20 Views
3D-R2N2	0.351	0.368	0.372	0.378	0.382	0.383	0.382	0.382	0.383
AttSets	0.395	0.418	0.426	0.430	0.432	0.444	0.445	0.447	0.448
Pix2Vox++	**0.436**	**0.452**	**0.455**	**0.457**	**0.458**	0.459	0.460	0.461	0.462
Ours	0.360	0.414	0.438	0.450	**0.458**	**0.470**	**0.477**	**0.480**	**0.483**

## Data Availability

The data presented in this study are available in ShapeNet at https://www.shapenet.org/, accessed on 11 January 2024. reference number [41]. ShapeNet is a publicly accessible dataset commonly used for 3D object reconstruction research. No new data were generated in this study, and the analysis was conducted using this publicly available dataset.
